# “Knowledge, clinical experience, and perceived need for training regarding molar-incisor hypomineralization among a group of Egyptian dental students: a cross-sectional study”

**DOI:** 10.1186/s12903-022-02356-2

**Published:** 2022-08-02

**Authors:** Alaa Mohammed Yehia, Amr M. Abdelaziz, Amira Badran

**Affiliations:** Faculty of Dentistry, Ain Shams University, Cairo, Egypt

**Keywords:** Molar incisor hypomineralization, Knowledge, Clinical experience, Need for training, Students, Post-eruptive enamel breakdown

## Abstract

**Background:**

Molar-Incisor Hypomineralization (MIH) is a common oral health condition that can lead to difficulties and complications for both dental professionals and patients. It also has a negative impact on the oral health-related quality of life. The present study aimed to assess the knowledge, clinical experience, and perceived need for training of a group of Egyptian dental students regarding MIH.

**Methods:**

Paper-based survey administration method was used to collect the responses of dental students regarding their knowledge, clinical experience, and perceived need for training about MIH. The survey consisted of two sections of questions regarding clinical features, etiological factors, prevalence, materials used in treating these teeth, factors affecting the choice of restorative materials, and their preferences regarding clinical training of MIH. Descriptive statistics was used for the data analysis by using SPSS® Statistics Version 26.

**Results:**

About two-thirds of the respondents were familiar with MIH (69.2%). The vast majority of students (87.8%) had difficulty distinguishing MIH as a developmental defect that differs from other tooth conditions (*p* < 0.001); most commonly enamel hypoplasia. The most common defects seen by the respondents were yellow/brown opacities (59.1%). Nearly half of the students (45.2%) choose composite resin as the material of choice for the treatment of MIH-affected teeth with aesthetics being the most common factor affecting the selection of restorative material. Almost all students expressed their needs for further clinical training on MIH, especially on treatment aspects.

**Conclusions:**

Most students are familiar with MIH theoretically. However, there is an urgent need to include clinical training on MIH diagnosis in the practical sessions of pediatric dentistry courses.

## Background

The term Molar Incisor Hypomineralization (MIH) was initially introduced in 2001 to describe a qualitative defect of the enamel mineralization affecting at least one of the first permanent molars (FPMs) with or without affection of the permanent incisors [1]. The prevalence of MIH worldwide has been found to be from 2.4 to 40% [2]. This wide range may be due to the use of different indices, diagnostic criteria, variability between examiners, lack of standardized methods of recording lesions and different age groups [3]. There are very few studies investigating MIH prevalence in Africa and the Middle East. However, two recent studies in Egypt reported MIH prevalence to be 2.3% in a group of Egyptian children that aged from 8 to 12 years in Cairo [4] and 9.98% among the same age group school children in Suez Canal sector cities [5].

Several possible etiological factors that might occur during the prenatal, perinatal or postnatal period can be the cause as proved by several studies [6, 7]. For instance, antibiotics usage during pregnancy or in the first year of life [8], illness during the first four years of life [6], urinary infection during the last trimester [9], genetic factors as well as environmental factors [10], and any traumatic birth event [9] have been found to have an association with MIH.

MIH has different clinical manifestations ranging from white to yellow–brown coloured enamel opacities with a demarcated line between the lesion and healthy enamel. The affected enamel can be sheared off under functional masticatory forces resulting in what is called post eruptive breakdown (PEB) [7, 11].

MIH burden has been found to be considerable where 878 million people across the world were MIH-affected and 17.5 million new cases are identified each year [12]. Additionally, MIH is considered one of the greatest challenges in dentistry because of its possible complications and difficulties in treating MIH-affected teeth as well as its negative impact on the oral health-related quality of life [13]. Therefore, early diagnosis of MIH by dental practitioners is considered the main step in the preventive approach of MIH management as recommended by the EAPD [14]. It is substantial to prevent complications such as hypersensitivity, poor aesthetics, rapid progression of dental caries, and first permanent molar (FPM) extraction in severe cases [15].

Unlike many other educational fields, dental education is based on three main components that form a triad: theoretical knowledge, laboratory practice, and clinical practice. These elements are highly essential to obtain a competent general dental practitioner who has sufficiently high-level clinical skills in diagnosing and treating different oral diseases [16]. MIH is one of the learning outcomes of both pediatric dentistry course and operative course in the undergraduate dental education program in most dental schools in Egypt because it is one of the public health concerns in dentistry. Therefore, assessment of dental students’ knowledge and clinical experience in different dental schools regarding previously mentioned aspects of such a condition is crucial. After the assessment, any areas of incompetence in either knowledge or clinical experience should be included in the undergraduate dental curriculum.

Previous similar studies have investigated knowledge, perceptions, and perceived need for training regarding MIH among dental students in different countries [17–21]. To the best of our knowledge, there is insufficient information regarding the knowledge and clinical experience of dental students about such condition in Egypt. Hence, the objective of this study is to assess the knowledge, clinical experience, and perceived need for training regarding MIH among a group of dental students who represent the future dental practitioners. Thus, assessing the effect of the growing awareness of the condition on dental curricula and the need for further training.

## Participants and methods

### Study design

The present cross-sectional study employed a survey to solicit responses from a group of dental students at the Faculty of Dentistry, Ain Shams University about their knowledge, clinical experience, and perceived need for training regarding MIH.

### Participants and sample

Students from two successive academic years (2019/ 2020) were invited to participate in the study after an approval of the administrative department of the Faculty. Fifth year dental students were selected because MIH is one of the topics that are taught during the pediatric dentistry course in this academic year. Ethical approval was sought from the research ethical committee at Faculty of Dentistry, Ain Shams University (FDASU-RecEM031818). Data were collected from January 2019 to January 2020.

Sample size calculation was performed utilizing the single proportion formula devised by Steven K. Thompson based on the research question regarding the prevalence of MIH knowledge. By adopting a confidence interval of (95%), a margin of error of (4%) with finite population correction and MIH knowledge prevalence of (76.9%) based on the results of a previous study [17]. The predicted sample size (*n*) was found to be (426) cases after adding 20% for non- responses and incomplete forms of the survey. A total population sampling technique which is a type of purposive sampling has been employed where the entire population who have the same characteristics (all fifth year dental students at Ain Shams University) were recruited in the study.

### Data collection tool

Paper-based survey administration method was used to collect the responses of the students as it was more convenient and it also ensured higher response rate. A validated structured questionnaire that had been used in previous studies [17, 19–23] was pilot tested on 20 students who were not included in the statistical analysis. After evaluating the responses, the questionnaire was considered appropriate to be used unmodified. It was self-administered in English language as dental students in Egypt receive their dental education in English.

The questionnaire consisted of a cover information sheet which included an invitation for participation, the aim of the study and definition of the condition under investigation. Images of hypomineralized FPMs & incisors were also provided in the cover sheet [24, 25]. Students were informed that participation in the present study is voluntary and anonymous with no effect on their course or their assessments if any of them decided not to participate, and that filling the questionnaire and returning it back to the principal investigator by putting it in a locked box with a thin opening at the top within one week will be considered as a written consent for participation.

The questionnaire consisted of 18 close-ended questions including either yes, or no questions or multiple-choice ones to allow for comparison with other studies. It consisted of two sections of questions, where the first section included questions regarding students’ familiarity with MIH, sources of information, knowledge of MIH clinical features, confidence in diagnosing MIH, difficulty in distinguishing MIH from other developmental defects, the frequency of encountering such defects, and their perception of MIH prevalence in their community. The second section included questions about MIH etiological factors, clinical applications regarding MIH such as the most commonly used restorative materials in treating these teeth, factors affecting their choice of restorative materials, the clinical problems reported in managing MIH affected teeth and their choices and perceptions regarding the need for clinical training on MIH.

### Statistical analysis

**Fig. 1:**
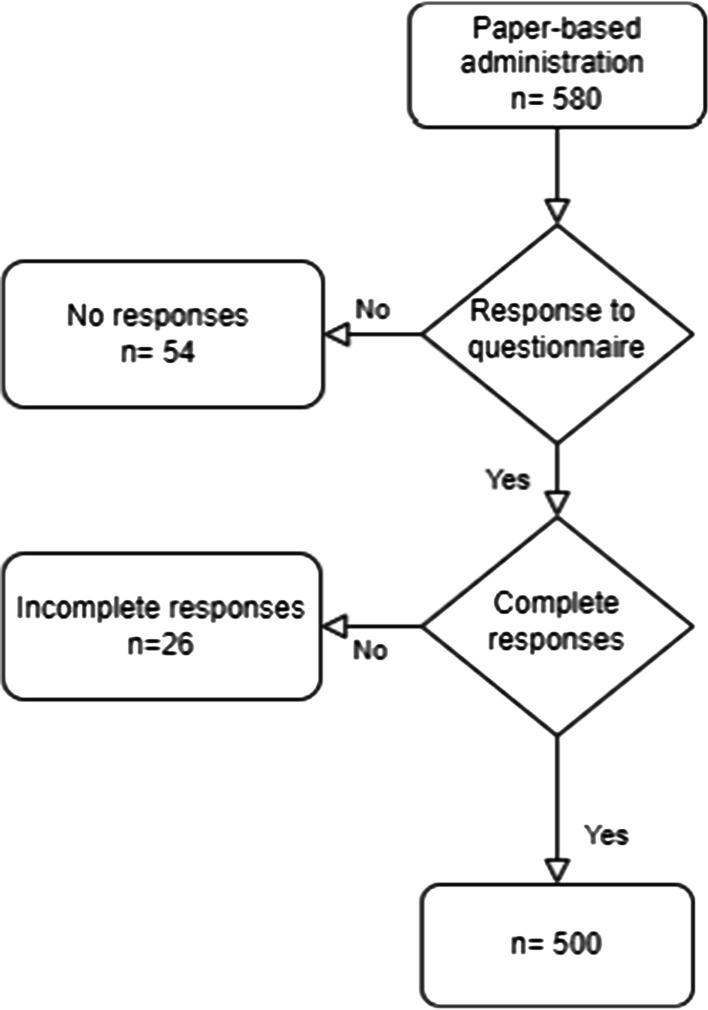
Flow chart of the number of participants included in the study

Descriptive statistics (simple frequency distributions) of students’ responses to each question regarding knowledge, clinical experience, and perceived need for training regarding MIH were produced. These data were analyzed using the fisher’s exact test followed by multiple pairwise comparisons with the z test with Bonferroni correction. Only completed questionnaires were included in the analysis. Out of 580 distributed questionnaires, 500 complete responses (318 females & 182 males) returned to the principal investigator with the overall response rate of 86.2% (Fig. 1).

The significance level was set at *p* ≤ 0.05 within all tests. Data were entered into a spreadsheet (Microsoft Excel) and then statistical analysis was performed with SPSS® Statistics Version 26.

## Results

As shown in (Table 1), about two-thirds of the dental students (69.2%) were familiar with MIH with significant difference between their perceived sources of knowledge. Nearly half of the students (46%) selected lectures as their source of information (*p* < 0.001). A statistically significant percentage of the students (87.8%) had difficulty distinguishing MIH from other developmental tooth conditions (*p* < 0.001); most commonly enamel hypoplasia.

**Table 1:** Knowledge, and clinical experience with respect to MIH diagnosis and prevalence

Questions		*N* (%)	*P*-value
Are you familiar with MIH? (yes)		346 (69.2%)	< 0.001*
If yes, how did you hear about it?	Dental Journals	7 (1.1%)	< 0.001*
	Lectures	230 (36.7%)#	
	Lecture notes	45 (7.2%)	
	Brochures or Pamphlets	4 (0.6%)	
	Internet	171 (27.3%)	
	Books	13 (2.1%)	
	Dental clinic/clinical supervisor	133 (21.2%)	
	Other students	22 (3.5%)	
	A friend was diagnosed with MIH	2 (0.3%)	
Do you know the clinical features of MIH? (yes)		309 (61.8%)	< 0.001*
Do you know if there are clinical criteria to diagnose MIH?	Yes, and I know how to implement them	16 (5.3%)	< 0.001*
	Yes, but I do not know how to implement them	227 (75.2%) #	
	No	59 (19.5%)	
In clinic, do you know if you can identify a patient with MIH? (yes)		297 (59.4%)	< 0.001*
How confident do you feel when diagnosing MIH?	Very confident	1 (0.3%)	< 0.001*
	Confident	42 (13.9%)	
	Slightly confident	207 (68.5%) #	
	Not confident at all	52 (17.2%)	
Do you have difficulty-distinguishing MIH as a developmental defect of enamel that differs from other tooth conditions? (yes)		439 (87.8%)	< 0.001*
If yes, which ones?	Dental fluorosis	134 (18.6%)	< 0.001*
	Enamel hypoplasia	382 (52.9%) #	
	Amelogenesis imperfecta	199 (27.6%)	
	Dentinogenesis imperfecta	7 (1.0%)	< 0.001*
How often do you notice these teeth in clinic?	Weekly basis	7 (2.3%) #	
	Monthly basis	151 (50.0%)	
	Yearly basis	144 (47.7%)	
Approximately what proportion of patients do you observe these teeth in?	< 10%	156 (52.0%)	< 0.001*
	10–25%	140 (46.7%)	
	> 25%	4 (1.3%) #	
Which of the following features do you most frequently notice regarding severity of the defect?	White demarcation	46 (15.3%)	< 0.001*
	Yellow/brown demarcation	178 (59.1%) #	
	Post-eruptive enamel breakdown	77 (25.6%)	
In clinic, have you encountered demarcated hypomineralized defects in permanent teeth other than the first permanent molars and incisors? (yes)		40 (13.3%)	< 0.001*
If yes, please name the tooth/teeth	Canines	25 (62.5%) #	< 0.001*
	Premolars	13 (32.5%)	
	Second permanent molar	2 (5.0%)	
How frequently do you notice demarcated hypomineralized lesions in the second primary molar tooth in comparison to the first permanent molar tooth?	More frequently	4 (1.3%)	< 0.001*
	Less frequently	152 (50.3%) #	
	The same as for the first permanent molar	5 (1.7%)	
	Never seen it	141 (46.7%)	
Are you aware of the prevalence of MIH in Egypt? (yes)		46 (9.2%)	< 0.001*
Do you think it would be worthwhile investigating the prevalence in Egypt? (yes)		447 (89.4%)	

#The reference answer in each question against which the significant difference has been tested

*significant (*p* ≤ 0.001)

More than half of the students (52%) reported identifying MIH in less than 10% of the patients they encounter in the clinic (Table 1). The most common defects seen by the respondents were yellow/brown opacities (59.1%). Most of the students (68.5%) felt slightly confident when diagnosing MIH with the majority of them (75.2%) acknowledged the presence of clinical criteria for MIH diagnosis as well as their inability to implement them (*p* < 0.001). Nearly half of them (46.7%) never seen Hypomineralized Second Primary Molars (HSPMs) in the clinic. Almost all students (90.8%) were not aware of the prevalence of MIH in Egypt.

As shown in (Table 2), most students have selected more than one possible etiological factor, which supports the common belief that MIH is a multifactorial condition. Generally, genetic factors were the most commonly identified etiological factor by the vast majority of students (89.4%) (*p* < 0.001). Composite resin has been selected as the material of choice for treatment of MIH-affected teeth with aesthetics being the most common factor affecting the selection of restorative material. Nearly all students (95%) suggested including clinical training regarding MIH in their dental courses; in particular, on the treatment aspects of MIH.

**Table 2:** Knowledge, and clinical experience regarding MIH etiology, management, and perceived need for training

Questions		*N* (%)	*P*-value
Which factor(s) do you think are involved in the etiology of MIH?	Genetic factors Chronic medical condition(s) that affect the mother during pregnancy Chronic medical condition(s) that affect the involved child Antibiotics/medications taken by the mother during pregnancy Antibiotics/medications taken by the involved child Environmental contaminants Acute medical condition(s) that affect the mother during pregnancy Acute medical condition(s) that affect the involved child Fluoride exposure	447 (28.5%) # 264 (16.9%) 105 (6.7%) 219 (14.0%) 79 (5.0%) 227 (14.5%) 103 (6.6%) 43 (2.7%) 79 (5.0%)	< 0.001*
Which material do you use MOST in treating MIH molars?	Amalgam Compomer Composite resin Resin Modified GIC Flowable composite resin Preformed crowns High Fluoride GIC Glass Ionomer Cement ICON	6 (1.2%) 21 (4.2%) 226 (45.2%) # 137 (27.4%) 5 (1.0%) 47 (9.4%) 48 (9.6%) 9 (1.8%) 1 (0.2%)	< 0.001*
Which factors influence your choice of restorative material?	Adhesion Remineralization potential Aesthetics Sensitivity Patient/parent preference Personal experience Durability Research findings	163 (21.8%) 108 (14.4%) 207 (27.7%) # 73 (9.8%) 28 (3.7%) 9 (1.2%) 132 (17.6%) 28 (3.7%)	< 0.001*
Do you think MIH is a clinical problem? (yes)		290 (96.3%)	< 0.001*
If yes, what do you experience problems with?	Diagnosis Aesthetics Achieving adequate local anesthesia Determining the restorative margins of affected enamel Providing adequate restorations Long-term success of restorations Achieving patient comfort (for function, oral hygiene)	115 (16.3%) 206 (29.3%) # 119 (16.9%) 59 (8.4%) 28 (4.0%) 159 (22.6%) 18 (2.6%)	< 0.001*
Would you suggest including clinical training regarding MIH in your dental course? (yes)		475 (95.0%)	< 0.001*
If yes, in which area(s) do you think you need to know/be taught about the most?	Diagnosis Etiology Treatment Prevention Complications	386 (36.8%) 216 (20.6%) 441 (42.0%) # 5 (0.5%) 1 (0.1%)	< 0.001*

#The reference answer in each question against which the significant difference has been tested

*significant (*p* ≤ 0.001)

## Discussion

The importance of this investigation is to highlight knowledge gaps that need to be addressed and to estimate the need for inclusion of MIH clinical training in the undergraduate curriculum. Results from this cross-sectional study showed that nearly two thirds (69.2%) of the dental students were familiar with MIH and that the most common sources of information for them were lectures and lecture notes, followed by the internet and clinical supervisors in the dental clinic. These findings are consistent with similar studies [18–21], but in contrast with a study in Saudi Arabia where 64% of the dental students had not heard about it [17].

Although more than half of the dental students in the present study reported having knowledge regarding the clinical features of MIH, however the vast majority (87.8%) of them were found to have difficulty distinguishing MIH from other developmental defects of enamel (DDE) especially Enamel Hypoplasia and Amelogenesis Imperfecta (AI). This could be explained by the fact that most of the MIH-related information in the undergraduate dental curriculum is theoretical either in the operative course or the pediatric dentistry course that are provided in the fifth year with little or no clinical exposure to such condition in the clinical training. This conclusion is also supported by another finding where more than two thirds (75.2%) of the dental students in the present study reported knowing the diagnostic criteria of MIH without knowing how to implement them clinically. These findings agreed with those of similar studies where most dental students had knowledge about the clinical features of MIH [18–20].

Similar to other studies, nearly half of the dental students reported encountering patients with MIH condition; nearly half of them reported encountering such cases on a monthly basis, about half reported yearly basis and rare selection of the weekly basis has been reported [17–19, 21]. This could represent their perceived estimation of MIH prevalence and reflect ostensible existence of this condition amongst the population which in turn signifies the necessity of conducting epidemiological surveys to provide reliable prevalence data. Additionally, these findings also highlighted the relatively low chance of dental students to encounter MIH. Therefore, the result in the present study must be interpreted with great caution.

About two-thirds of the dental students reported the yellow–brown opacities as the most frequently observed defects. This has been interpreted by other studies based on the fact that such type of lesion could be the least frequently confused with alternative diagnoses such as fluorosis, enamel hypoplasia and carious white spot lesions [18–23, 26]. PEB was less frequently encountered in the surveyed population. This might be explained by the fact that PEB is masked by extensive caries or atypical restoration as reported by previous research [14].

The vast majority of dental students (90.8%) were not aware of the prevalence of MIH in Egypt where a relatively high percentage of them over estimated MIH prevalence as being either from 10 to 25% or > 25% which is not agreed with MIH prevalence in Egypt according to the literature [4, 5]. This might be a result of the dental students’ misdiagnosis of other developmental conditions like enamel hypoplasia, AI or fluorosis as MIH. Thence, not surprisingly, an overwhelming majority of them (89.4%) recommended that investigating the prevalence of MIH in Egypt would be worthwhile. These results support those in similar studies which have reported that practitioners are uncertain about the prevalence of MIH in their communities [19–21, 27].

Similar to previous studies, nearly half of the students in the present study reported never seeing MIH-like defects in the second primary molars [18, 19, 21]. The other half reported observing MIH-like defects in the second primary molar less frequently than in FPMs.

Consistent with other studies [17–21], most respondents 68% selected more than three possible etiological factors with one of the most frequently mentioned answers was "genetic factors". This is supported by previous literature which illustrated the synergistic action between genetic factors, external stimuli, systemic and medical factors [10]. Chronic maternal diseases during pregnancy and environmental contaminants, were frequently selected as etiological factors, consistent with the studies reported that medical problems during pregnancy are more common in mothers of MIH-children than in mothers whose children do not have MIH [28, 29]. Fluoride has been also implicated by a number of participants as a possible etiological factor which may indicate there is still confusion between fluorosis and other developmental enamel defects among dental students.

The wide variety of materials that can be used in restoring MIH-affected teeth may reflect the lack of a comprehensive guide for therapeutic management. In the present study, the most preferred dental material used by dental students was composite resin, followed by RMGIC. These findings were consistent with several studies [17, 19–21, 30], but not in agreement with the results of other studies which reported GIC to be the preferred material [23, 24, 27]. Consistent with previous studies, esthetics, adhesion and durability were the most common factors influencing restorative material choice in the present study [19–21, 31].

Consensus that MIH is a clinical problem associated with clinical difficulties has been reflected in the responses of dental students who reported several problems experienced with MIH, most commonly esthetics, longevity of restorations, followed by sensitivity and difficulty achieving local anaesthesia, and difficulty providing long lasting restorations that might be due to the poor bond strength of different restorative materials to the affected enamel [32, 33]. These responses come in agreement with those of previous studies [24, 34, 35].

In the present study, almost all dental students expressed interest for including clinical training regarding MIH especially on treatment and diagnosis in undergraduate dental courses. This can help them to be aware about the significant challenges involved in managing patients with MIH and to decide on the best treatment options [17–21]. Lack of clinical experience and low rate of diagnostic confidence among participants can elucidate their request for training in the diagnosis as well.

### Study limitations

The most apparent shortcoming in the present study is the selectivity of the sample population which is limited to dental students at a single university, thus it does not reflect current MIH knowledge in general. Additionally, concerning the self-reported nature of the study, there is a possibility of response biases because of the social desirability that may have led respondents to over report their knowledge and perceptions [36]. Nevertheless, the present study provides a reference database for future broader surveys in the Middle East and guidelines for including both theoretical information and clinical training on MIH in the undergraduate dental curriculum.

## Conclusion

Although most dental students were familiar with MIH theoretically, the majority of them were unconfident when diagnosing MIH and had difficulty distinguishing MIH from other developmental enamel defects. Thus, an imperative need to include clinical training on the diagnosis and management of MIH in the undergraduate courses should be considered. Otherwise, those students who represent the future dentists will face similar challenges in terms of managing patients with MIH.

## Data Availability

Data are available from the corresponding author upon reasonable request. All authors had full access to all data in the study and take responsibility for the integrity of the data.

## Abbreviations

MIH: Molar incisor hypomineralization
FPMs: First permanent molars
PEB: Post eruptive breakdown
SPSS: Statistical package for social science
HSPM: Hypomineralized second primary molars
DDE: Developmental defects of enamel
AI: Amelogenesis imperfecta
